# Progress in Surgery

**Published:** 1899-11-25

**Authors:** 


### PROGRESS IN SURGERY.

### HERNIA.

**The Radical Cure of Inguinal Hernia.**—C. A. Sturrock[1] advocates the following operation. Exposure of the sac by a short incision, with its centre over the external abdominal ring. Isolation of the exposed sac from its attachments up to the internal abdominal ring, care being taken to avoid division of any of the muscles which form the anterior wall of the inguinal canal. Then an incision is made into the lowest part of the sac, and through this incision either the forefinger or a forceps is passed into the abdominal cavity; the tip of the finger or forceps is then cut down upon at a point one inch above the spine of the pubes, and midway between the internal and external borders of the rectus abdominis. Through this incision a forceps is passed and carried down through the sac. The edges of the incision in the sac are grasped by the instrument. The sac of the hernia is then pulled through the incision in the rectus. It becomes, of course, inverted. The aperture through which the hernia escaped is then closed. The redundant portion of the sac is now ligatured and excised, and a stitch inserted into the rectus which includes the stump of the sac. Another stitch, which includes the fascia of the rectus, closes the incision in the skin. In femoral hernia the second incision may be made at the same point. It is remarked that ventral hernia is unknown in the region where the second incision is made, and the weak spot made there will probably have no ill effect. The author wrote his paper in ignorance of Kocher's advocacy of a similar mode of inverting the sac. Kocher, however, draws out the sac in a position 2 cms. external to the internal abdominal ring, perhaps a position less advantageous than Sturrock's.

**Hernia of the Vermiform Appendix.**—W. H. Battle[2] records another example of this condition in a man of 73. The patient was admitted into St. Thomas's Hospital with an irreducible scrotal hernia, evidently omental, and with a collection of fluid below it. As the man could not wear a truss, and the rupture remained irreducible after a prolonged rest in bed, an operation for radical cure was performed. The sac was found occupied by adherent omentum, below which was a collection of fluid constituting a hydrocele of the hernial sac. The appendix was found in the thickened omentum, and was adherent. This was divided in the usual manner, and the omentum divided in strands. The canal was closed by Bassini's operation, and recovery was uneventful. There had been catarrhal inflammation of the appendix, but probably the protrusion had occurred after this, the surrounding of the appendix by the omentum having prevented serious peritonitis. In both femoral and inguinal hernia the vermiform appendix is usually accompanied by the cæcum, and is rarely found by itself, as in this case. Mr. Chavasse[2] also relates a case of hernia of the appendix. The patient was a woman of 65, with a large family She had had a right femoral rupture for some years. It was operated upon because it had become irreducible, and the sac found to be thick-walled and old, and to contain a much congested, thick, and coiled-up appendix, and also about six drachms of dark-coloured serous fluid. Chavasse thinks the case bears out the suggestion of McAdam Eccles, that the inflammation of the appendix in these cases often occurs after its prolapse into the hernial sac, and that strangulation is secondary, being caused by the acute inflammatory processes taking place in a confined space. Mr. Steele[3] has recorded a case of strangulation of the cæcum and vermiform appendix in a right inguinal hernia. The patient was a child six weeks old, and a history was given of three days' vomiting. The hernia proved to be a congenital one of the cæcum and vermiform process. Steele thinks, from the history of the case, that while the bowel contents remained fluid they could pass on, but when the cæcum became filled with solid fæces, as it was found to be at the operation, the hernia first became incarcerated, and then slowly strangulated. Strangulation of hernia is rare in young children, and slow in occurring, probably because the inguinal rings are so soft in early life. A radical cure was successfully performed in this case, though there was difficulty in reducing the bowel because the mæso-cæcum was adherent to structures external to the ring.

**Laparotomy for Strangulated Hernia.**—Mr. Henry

Smith[7] records the case of a fellah, operated upon at the American Mission Hospital in Upper Egypt for strangulated right inguinal hernia, apparently produced by lifting a heavy stone a few hours previously. Dr. McLaughlin operated. There were seven or eight inches of congested bowel in the sac, and the most painstaking manipulations, with inversion of the patient and free enlargement of the incisions, failed to effect reduction of the coil. Median laparotomy was therefore done, and one hand passed into the abdomen. Flatus was passed along the bowel by the movements of this hand, and finally by working with both hands the bowel was partly pushed and partly drawn back into the abdomen. Recovery without a bad symptom resulted.

**The Preventive and Curative Treatment of Post-Operative Ventral Hernia.**—Dr. W. T. Bull[4] recently brought before the Practitioners' Society of New York a case of ventral hernia 14 years after abdominal section. The operation was for penetrating wound of the abdomen from a pistol bullet. Four perforations in a length of seven inches of intestine had been sutured. It may be incidentally noted that this suturing diminished the lumen of the bowel by a half, and yet the functions of the intestine were not interfered with. The man had resumed his work, that of a truckman, three months after leaving hospital, and had been engaged in it ever since. He had worn six imperfect and cheap abdominal belts during this period. In the centre of the scar a ventral hernia of very moderate size existed. The interest of the case was that, though the patient had persisted in a laborious occupation, and though the incision was a very long one, and healed by granulation, yet the hernia was of very moderate size.

A number of drainage tubes had been used, and there must have been a considerable amount of fibrinous peritonitis, but this had not caused any symptoms. Dr. Bull said it was a fact that the largest and most voluminous ventral herniæ were seen in people who did not use their abdominal muscles, or in women whose muscles had been weakened by inactivity and childbirth. This indicated that proper exercise of the muscles should be encouraged rather than restricted. In the discussion on the case Dr. McBurney expressed the view that false or new tissue had formed within the abdomen, preventing the intestines from bearing very strongly against the cicatrix. He still thought it a wise precaution to instruct these patients not to make violent efforts.

Dr. A. B. Johnson[5] describes a new method he used in attempting to cure a ventral hernia following an operation for appendicitis performed three years before. The scar was flat when the woman was supine, but when she stood up projected, forming a tumour measuring three by four inches. Through the scar an orifice in the abdominal wall was felt three inches long, with hard edges. In the operation on the outer side the external oblique was dissected away from the underlying structures for two inches outwards from the edge of the orifice. On the inner side the sheath of the rectus was opened. The posterior layer of the rectus sheath and peritoneum upon the inner side were united to the internal oblique, and transversalis and peritoneum on the outer side. The body of the rectus muscle was then displaced outwards from its sheath, and by mattress sutures united to the under surface of the aponeurosis of the external oblique. Thus this layer of muscle lay in front of the first row of buried sutures. Then the edge of the external oblique was sutured to the anterior layer of the rectus sheath. No support was worn, and at the end of five months no weakness was evident. It was difficult to say positively whether the rectus had remained in its new position or not, owing to the extreme thickness of the abdominal wall.

G. M. Edebohls[6] has written a paper on the best modes of operating for appendicitis in order to avoid subsequent hernia, and to diminish the period of confinement to bed after the operations. He concludes that hernia need no longer be dreaded after these operations, whether for acute or chronic appendicitis, with or without pus. Where there is no drainage required, he states that confinement to bed need not be for longer than a week; and, in suppurative cases, unless a fæcal fistula form, the period of confinement need rarely exceed a fortnight. The mode of incising the fascia and muscles is of the most importance. The incision should be Battle's ("trap-door"), through the rectus abdominis, when that lies sufficiently over the swelling. If not, it should be McBurney's "grid-iron" incision. The only difficulty is with the cases requiring drainage. For these McBurney's incision is usually required. For the drainage gauze is used, passing straight to the infected region or regions, and no sutures applied. As soon as possible these are dispensed with, this being from the fifth to the seventh day. The cavity the plug occupied rapidly fills, and the granulations reach the level of the bottom of the abdominal incision. Then an anæsthetic is given, and the granulating edge of the wound dissected off in one clean piece from skin and fat, and the other layers of the abdominal wall. The incision then becomes identical in every particular with the freshly-made grid-iron incision, and is dealt with accordingly. On the next or the third day the patient bids adieu to his bed. Edebohls concludes by stating that there seems no good reason why all patients suffering from appendicitis, acute or chronic, should not have the benefit of operation.

[1] Edinburgh Med. Jour., Oct. [2] Lancet, June, 1899. [3] Ditto, July, 1899. [4] Med. Rec., April, 1899. [5] Annals of Surgery, May, 1899. [6] Med. Rec., May, 1899. [7] Lancet, Aug., 1899.

